# Three-year outcomes and predictors for full recovery in patients with early-stage psychosis

**DOI:** 10.1038/s41537-022-00301-4

**Published:** 2022-10-27

**Authors:** Ling Li, Fatima Zahra Rami, Bo Mi Lee, Woo-Sung Kim, Sung-Wan Kim, Bong Ju Lee, Je-Chun Yu, Kyu Young Lee, Seung-Hee Won, Seung-Hwan Lee, Seung-Hyun Kim, Shi Hyun Kang, Euitae Kim, Young-Chul Chung

**Affiliations:** 1 Department of Psychiatry, Jeonbuk National University Medical School, Jeonju, Republic of Korea; 2 Research Institute of Clinical Medicine of Jeonbuk National University-Biomedical Research Institute of Jeonbuk National University Hospital, Jeonju, Republic of Korea; 3 Department of Psychiatry, Chonnam National University Medical School, Gwangju, Republic of Korea; 4 Department of Psychiatry, Inje University Haeundae Paik Hospital, Inje University College of Medicine, Busan, Republic of Korea; 5 Department of Psychiatry, Eulji University School of Medicine, Eulji University Hospital, Daejeon, Republic of Korea; 6 Department of Psychiatry, Eulji University School of Medicine, Eulji General Hospital, Seoul, Republic of Korea; 7 Department of Psychiatry, Kyungpook National University School of Medicine, Daegu, Republic of Korea; 8 Department of Psychiatry, Inje University College of Medicine, Goyang, Republic of Korea; 9 Department of Psychiatry, Korea University College of Medicine, Guro Hospital, Seoul, Republic of Korea; 10 Department of Social Psychiatry and Rehabilitation, National Center for Mental Health, Seoul, Republic of Korea; 11 Department of Psychiatry, Seoul National University Bundang Hospital, Seongnam, Republic of Korea

**Keywords:** Psychosis, Schizophrenia

## Abstract

In the present study, various outcomes over 3-year period in patients with early stage psychosis including remission, recovery, relapse and medication adherence were investigated. Predictor for full recovery at year 3 was also examined. Three-year follow-up data in 534 patients with schizophrenia spectrum disorders (SSD) and psychotic disorder not otherwise specified (PNOS) were examined for overall outcome trajectories. The data of completers at year 3 (*n* = 157) were used to identify predictors for recovery using logistic regression. The rates of symptomatic remission and full recovery at 6-, 12-, 24-, and 36-month follow-up were 76.10, 69.20, 79.50, and 79.10%, and 22.80, 26.40, 28.60, and 39.60%, respectively. The rates of drop-out and relapse at 6-, 12-, 24-, and 36-month follow-up were 25.4, 29.5, 38.6, and 51.1%, and 3.7, 8.9, 19.0, and 38.9%, respectively. The rates of good adherence and prescription of Long-Acting Injectable Antipsychotics (LAIA) at 6-, 12-, 24- and 36-month follow-up were 87.8, 88.0, 91.9, and 93.9%, and 18.3, 21.7, 22.0, and 25.5%, respectively. Significant predictors for full recovery were duration of untreated psychosis (DUP), family intimacy and physical activity. We observed similar or better results on remission, recovery, and relapse rates compared to other previous studies. Effective psychosocial intervention should be provided to shorten the gap between remission and recovery rates and to address DUP, family issues, and exercise to enhance recovery.

## Introduction

To improve recovery and remission rates in early-stage psychosis, it is important to understand its trajectory including remission, recovery, and relapse especially after first episode psychosis (FEP). Early-stage psychosis is usually defined as a clinical diagnosis within < 2 or 5 years of psychotic illness^1–3^ and ≤ 2 years of antipsychotic treatment in the present study. FEP is defined as patients who had received < 2~16 weeks^4,5^, 6 months^6^, or 12 months^7,8^ of antipsychotic treatment. As there is a paucity of studies on outcomes with early-stage psychosis, literature on FEP will be reviewed and discussed with regard to the results of the present study.

One systematic review reported a mean symptomatic remission rate of 36% (range: 17–78%, with a follow-up duration of 6 months to 7 years) in patients with FEP^9^. The most recent systematic review and meta-analysis^10^ reported even higher rates, with a mean remission rate of 58% (53–63%) during a mean follow-up of 5.5 years. Jääskeläinen^11^ reported a 17% (9–20%) recovery rate, whereas Lally^10^ reported a mean recovery rate of 38% (30–46%) over a mean follow-up of 7.2 years. A thorough understanding of the predictors of remission and recovery in FEP is also crucial for implementing mental health service systems. Predictors of symptomatic remission in FEP include better premorbid functioning^9^, milder symptoms at baseline^9,12,13^, early response to treatment^9,12,14^, and a shorter duration of untreated psychosis (DUP)^9,12,14,15^. As for the predictors of recovery, similar effects have been reported for better premorbid adjustment^16–19^ and a shorter DUP^12,14,15,17,18^. Other factors include being female^20^, better premorbid adjustment^16–19^, higher levels of education^12,14,19^, better neurocognition^15,21^, premorbid IQ^22^, superior occupational status^14,23^, being married^24^, adherence to medication^17^, favorable personality^25^, and fewer negative symptoms at baseline^12,16,17,20^.

Psychotic relapse can have devastating consequences including worsening of symptoms, progressive cognitive deterioration, impaired functioning^26,27^, and an increased burden for caregivers^28^. For young people with psychotic disorders, these relapse events undermine any sense of hope or optimism for the future and adversely affect their long-term psychosocial development^29^. Evidence indicates that the prevalence of a relapse of positive symptoms following treatment for FEP or first episode schizophrenia (FES) is 28%, 43%, 54%, and 80% at the 1-, 1.5–2-, 3-, and 5-year follow-up, respectively^15,30^. One meta-analysis reported that medication non-adherence, persistent substance use disorder, critical comments by caregivers, and poorer premorbid adjustment increased the risk for relapse 4-fold, 3-fold, 2.3-fold, and 2.2-fold, respectively^30^. Given the discontinuation rates for oral antipsychotics in patients with FES of 42% at 12 months^31^, the use of long-acting injectable antipsychotics (LAIA) could be a valuable option to prevent medication discontinuation and subsequent relapse. However, despite the clear advantages of using LAIA on clinical outcomes in FES, the prevalence of using LAIA after first hospitalization for SZ is actually quite low, at 8–10%^32^.

The Korea Early Psychosis Study (KEPS) is a prospective naturalistic observational cohort study involving patients with early-stage psychosis. The project took place from December 2014 to December 2021, and 11 hospitals participated. This was the first long-term prospective cohort study in Korea. It focused on overall outcome trajectories, including the rates of remission, recovery, drop-out, relapse, medication adherence, and prescription frequency of LAIA. Given that the prevalence of self-reported childhood abuse is common (26–39%) among patients with psychosis^33^ and rumination is associated with various psychopathologies in psychosis^34–36^, we were particularly interested in examining the impact of childhood trauma and rumination on outcomes in year 3 among patients with early-stage psychosis. We also narrowed our focus to schizophrenia spectrum disorders (SSD) and psychotic disorder not otherwise specified (PNOS), more homogenous groups. The aims of the present study were to identify various outcomes over a 3-year period in patients with early-stage psychosis, including remission, recovery, relapse, and medication adherence. We also examined predictors for full recovery at year 3 using logistic regression.

## Methods

### Participants

**Fig. 1:**
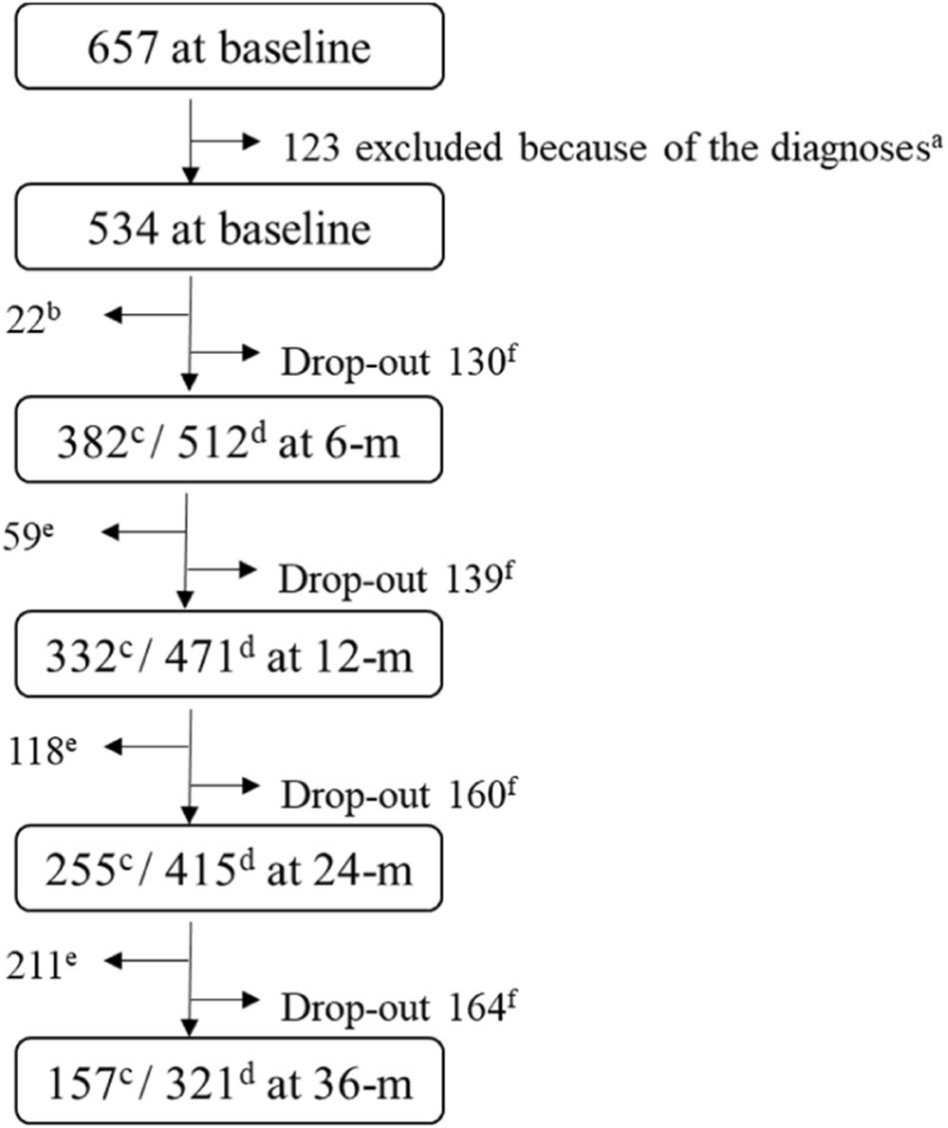
Flowchart illustrating numbers of subjects assessed or dropped out at each follow-up. ^a^Include schizoaffective disorder, delusional disorder and brief psychotic disorder. ^b^Subjects registered ≤6-m ago. ^c^Completer. ^d^Subjects registered at least 6-, 12-, 24-, or 36-m ago. ^e^Cumulative number of subjects registered ≤12-, 24- or 36-m ago plus subjects once dropped out and reentered the study. ^f^Cumulative number of drop-out (follow-up loss 118, withdrawal of consent 41 and death 5).

The subjects enrolled in the current study were from the KEPS. We used data collected from January 2015 to July 2020. At the time of analysis, 657 patients with early-stage psychosis had enrolled. Early-stage was defined as the duration of adequate antipsychotic treatment of more than 4 weeks but less than or equal to 2 years. We restricted analysis to individuals diagnosed as having SSD (schizophrenia [SZ] and schizophreniform disorder [SZFD]) and PNOS. Ultimately, 534 patients with early-stage psychosis were included in the study; notably, these patients had different durations of follow-up from registration (for details, see Fig. 1). Diagnoses were established using the criteria of the Diagnostic and Statistical Manual of Mental Disorders fourth edition^37^ and the Korean version of the Mini-International Neuropsychiatric Interview^38^. Two experienced psychiatrists from each institute participated in the diagnostic evaluation and reached a consensus on final diagnosis through discussion. All participants provided written informed consent in accordance with the protocol approved by the Ethics Committee of Jeonbuk National University Hospital and other participating hospitals (approval number CUH 2014-11-002).

### Assessments

#### Demographic and clinical data

Sociodemographic data (age, sex, education, type of medical insurance, and job type) were obtained at baseline. A family history of psychotic disorders, duration of untreated psychosis (DUP), duration of illness (DI), the Positive and Negative Syndrome Scale (PANSS)^39^, Clinical Global Impression (CGI) scales^40^, Social and Occupational Functioning Assessment Scale (SOFAS)^41^, the Calgary Depression Scale for Schizophrenia (CDSS)^42,43^, the Columbia-Suicide Severity Rating Scale (C-SSRS)^44^, and comorbid mental disorders were evaluated. DUP was defined as the interval between the onset of psychotic symptoms and initiation of antipsychotic treatment or hospitalization for psychosis. Clinical data on psychopathology were obtained at baseline, and at 2, 6, 9, 12, 18, 24, 30, and 36 months, with the exception of the CDSS and C-SSRS, which were evaluated at baseline and at 6, 12, 24, and 36 months. To ensure inter-rater reliability between the sites for the PANSS, SOFAS and C-SSRS, psychiatrists with more than 3 years of experience in this field participated in the ratings process and several workshops were held during the recruitment period.

#### Self-rated variables

This was the first prospective observational study in Korea, so we had no a priori variables of interest except for childhood trauma, and included variables based on a review of the literature on recovery. The scales surveyed at baseline were the Big Five Inventory (BFI-10)^45–48^, Brief Core Schema Scales (BCSS)^49,50^, Brief Resilience Scale (BRS)^45,46,51^, Brooding Scale (BS)^52^, Diet History Questionnaire (DHQ)^53,54^, Early Trauma Inventory Self Report-Short Form (ETISR-SF)^55^, Family Adaptability and Cohesion Evaluation Scales III (FACES-III)^56,57^, Family Intimacy (FI, 5-point Likert scale), Physical Activity Rating (PAR)^58,59^, and the Korean version of the Subjective Well-being Under Neuroleptics-Short Form (K-SWN)^60,61^. The BS consisting of 11 items measures the degree of rumination about past negative events. The DHQ score is categorized as poor (20–49), usual (50–79), or good (80–100).

#### Definitions of remission, recovery, drop-out, and relapse

The operational definition of symptomatic remission was a score of ≤3 on eight items of the PANSS (P1, P2, P3, N1, N4, N6, G5, and G9)^62^. The criteria for full recovery were based on those of previous studies^15,63–65^. These were (a) symptomatic recovery: score of ≤2 on the eight items of the PANSS; and (b) functional recovery: adequate social interaction (at least two meetings with a familiar person per month) and occupational functioning (having a job for more than 1/2 of the total duration, attending school regularly, or competence in the homemaker role). A duration of remission was 2 and 6 months at the 6-month follow-up and 12-, 24-, and 36-month follow-ups, respectively. For a full recovery, the remission duration was 2-, 6-, and 12-months at the 6-month, 12-month, and 24- and 36-month follow-ups, respectively. Drop-out was defined as missing two consecutive visits to the outpatient clinic. Relapse was defined as an exacerbation of symptoms ≥2 months after remission, such as psychiatric hospitalization, a CGI-S score ≥ 4 with an increase of ≥2, a CGI-I score ≥ 6 (much worse), a score of ≥4 for a psychosis item (P1, P2, P3, and P6) with an increase of ≥2, a ≥ 25% increase in the total PANSS score or a ≥ 10-point increase if the baseline score was ≤40, deliberate self-injury, clinically serious suicide or homicide ideation, a suicide attempt, or violent behavior resulting in significant injury to another person or property^66^. Patients were considered to have had a relapse if the re-emerged symptoms lasted for at least 1 week. To ensure inter-rater reliability for the PANSS and outcome evaluation among the sites, psychiatrists with more than 2 years of experience in this field participated in the rating process, and several workshops were held during the recruitment period. Medication adherence, prescription frequency of LAIA, and rates of accessing the mental health welfare center were also evaluated.

### Statistical analysis

The baseline data of subjects who were followed up to the 3-year versus those that had dropped out were compared using chi-square or independent *t*-tests, as appropriate. We estimated rates of remission and full recovery at 6-, 12-, 24- and 36-month follow-up for total subjects as well as subgroups with SZ, SZFD or PNOS. For logistic regression, the baseline data of the completers at 3-year follow-up (*n* = 161) were used to identify the predictors of a full recovery at year 3 in total subjects as well as subgroups with SZ, SZFD or PNOS. The demographic and clinical variables were tested initially using univariate regression analysis; variables with p-values of *p* ≤ 0.10 were then further evaluated using stepwise regression. All analyses were performed in R (version 3.4.0; R Core Team) and R Studio (version 1.0.143; R Foundation for Statistical Computing, Vienna, Austria). Significance was set to an alpha level of 0.05, and all *p*-values were two-sided.

## Results

**Fig. 2:**
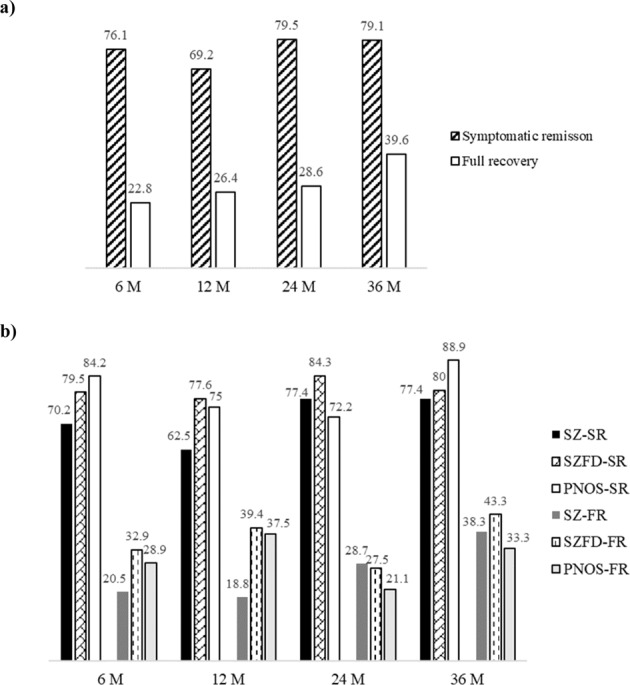
Rates (%) of outcomes over 3-year follow-up. Symptomatic remission (SR) and full recovery (FR) in **a** total participants and **b** in patients with schizophrenia (SZ), schizophreniform disorder (SZFD) or psychotic disorder NOS (PNOS).

Table 1 lists the demographic and clinical characteristics of the 534 subjects. Figure 1 presents the number of completers/ to be followed up and the drop-out at each follow-up point. The rates of symptomatic remission at the 6-, 12-, 24-, and 36-month follow-up in total subjects were 76.10, 69.20, 79.50, and 79.10%, and rates of full recovery at the same time points were 22.80, 26.40, 28.60, and 39.60% (Fig. 2 and Table S1). In the subgroup analysis, remission and recovery rates for SZFD and PNOS were higher in most of the follow-ups compared to those for SZ (Table S1). The rates of drop-out and relapse at the 6-, 12-, 24-, and 36-month follow-ups in total subjects were 25.4, 29.5, 38.6, and 51.1%, and 3.7, 8.9, 19.0, and 38.9%, respectively (Table 2). In subgroup analysis, the drop-out rates for PNOS were higher at all follow-ups compared to SZ and SZFD. The relapse rates for SZFD and PNOS were higher at later follow-ups (24- and 36-month) compared to SZ. The rates of good adherence and metabolic syndrome at the 6-, 12-, 24-, and 36-month follow-ups in total subjects were 87.8, 88.0, 91.9, and 93.9%, and 12.8, 17.4, 22.0, and 11.5%, respectively (Table 3). In subgroup analysis, PNOS showed lower rates of good adherence at all follow-ups compared to SZ and SZFD. The prescription rates of LAIA were 9.7% at baseline and 18–26% at follow-ups. Rates of accessing the mental health welfare facilities at baseline and at 6-, 12-, 24-, and 36-month were 7.4, 12.4, 11.0, 10.4, and 12.1%, respectively (Table 4).

**Table 1:** Demographic and baseline clinical characteristics of participants (*n* = 534).

	*n*	Mean ± SD
Gender	534	
male		208 (39.0)
female		326 (61.0)
Age, years	534	28.4 ± 8.5
Education	534	
Elementary school		5 (0.9)
Middle school		205 (38.4)
University		324 (60.7)
Type of medical insurance	534	
Health insurance		508 (95.1)
Near poor		4 (0.7)
Medicaid		22 (4.1)
Job type	533	
Unemployment		219 (41.1)
Non-professional		289 (54.2)
Professional		25 (4.7)
Family history of psychosis	534	
No		460 (86.1)
Yes		74 (13.9)
DUP, months	533	14.1 ± 26.3
DI, months	534	21.2 ± 29.6
Diagnosis	534	
Schizophrenia		367 (68.7)
Schizophreniform disorder		101 (18.9)
Psychotic disorder NOS		66 (12.4)
Comorbid mental disorder	534	
No		464 (86.9)
Yes^a^		70 (13.1)
PNASS	534	
Positive		18.4 ± 7.4
Negative		17.5 ± 7.1
General		37.9 ± 12.5
Total		73.8 ± 24.2
CDSS, total score	531	5.3 ± 4.8
C-SSRS		
Concreteness of suicidal ideation	197	2.7 ± 1.3
Intensity of suicidal ideation	194	2.5 ± 1.1
SOFAS	533	56.5 ± 13.5
BCSS, mean	505	
Self		1.7 ± 0.8
Others		1.8 ± 0.7
BFI-10, mean	502	
Extraversion		2.9 ± 0.8
Agreeableness		3.4 ± 0.7
Conscientiousness		3.1 ± 0.8
Neuroticism		2.9 ± 0.8
Openness		3.5 ± 0.9
BRS, mean	505	2.8 ± 0.8
BS, total	506	15.3 ± 8.4
DHQ, total	500	66.1 ± 15.1
20–49		74 (14.8)
50–79		325 (65)
80–100		101 (20.2)
ETISR-SF	506	
General trauma		1.7 ± 1.9
Physical abuse		1.8 ± 1.7
Emotional abuse		1.8 ± 1.8
Sexual abuse		0.6 ± 1.1
Total		5.9 ± 4.9
FACES-III, total	468	61.0 ± 13.5
FI, mean	458	3.6 ± 0.9
KmSWN, total	503	60.4 ± 18.9
PAR	505	
Frequency		2.6 ± 2.4
Intensity		1.7 ± 1.8

*BCSS* Brief Core Schema Scales, *BFI-10* Big Five Inventory, *BRS* Brief Resilience Scale, *BS* Brooding scale, *CDSS* Calgary Depression Scale for Schizophrenia, *C-SSRS* Columbia-Suicide Severity Rating Scale, *DI* Duration of illness, *DHQ* Diet History Questionnaire, *DUP* duration of untreated psychosis, *ETISR-SF* Early Trauma Inventory Self Report-Short Form, *FACES-III* Family Adaptability and Cohesion Evaluation Scales III, *FI* Family Intimacy, *KmSWN* Korean modification of Subjective Well-being Under Neuroleptics-Short Form, *m* months, *NOS* not otherwise specified, *PANSS* Positive and Negative Syndrome Scale, *PAR* Physical Activity Rating, *SOFAS* Social and Occupational Functioning Assessment Scale.

^a^No substance use disorders.

**Table 2:** Cumulative rates (%) of drop-out and relapse in follow-up time.

	6 m	12 m	24 m	36 m
Drop-out				
Total^a^	25.4 (130/512)	29.5 (139/471)	38.6 (160/415)	51.1 (164/321)
Schizophrenia	24.6 (87/354)	29.6 (95/321)	37.8 (111/294)	50.6 (120/237)
Schizophreniform disorder	21.1 (20/95)	25.5 (24/94)	37.0 (30/81)	45.5 (25/55)
PNOS	36.5 (23/63)	35.7 (20/56)	47.5 (19/40)	65.5 (19/29)
Relapse^b^				
Total^a^	3.7 (14/382)	8.9 (30/336)	19.0 (50/263)	38.9 (68/175)
Schizophrenia	3.4 (9/267)	8.3 (19/229)	14.9 (28/188)	32.0 (41/128)
Schizophreniform disorder	2.7 (2/75)	11.3 (8/71)	31.5 (17/54)	58.3 (21/36)
PNOS	7.5 (3/40)	8.3 (3/36)	23.8 (5/21)	54.5 (6/11)

*PNOS* Psychotic disorder not otherwise specified, *m* months.

^a^Total participants with schizophrenia, schizophreniform disorder or PNOS.

^b^Those who were dropped out without relapse were not counted but those who were dropped out without relapse and re-followed up were counted for denominator.

**Table 3:** Rates (%) of medication adherence and LAIA and its doses (mean ± SD mg) over 3-year follow-up.

	Baseline	6 m (*n* = 382)	12 m (*n* = 332)	24 m (*n* = 255)	36 m (*n* = 157)
Adherence					
Good (≥80)					
Total^a^		87.8 (318/362)	88.0 (271/308)	91.9 (217/236)	93.9 (138/147)
Schizophrenia		91.7 (232/253)	90.1 (192/213)	94.0 (157/167)	94.7 (107/113)
Schizophreniform disorder		81.9 (59/72)	90.6 (58/64)	92.0 (46/50)	92.3 (24/26)
PNOS		73.0 (27/37)	67.7 (21/31)	73.7 (14/19)	87.5 (7/8)
Fair (50~79)					
Total^a^		7.5 (27/362)	5.5 (17/308)	3.0 (7/236)	2.7 (4/147)
Schizophrenia		4.3 (11/253)	4.7 (10/213)	1.8 (3/167)	3.5 (4/113)
Schizophreniform disorder		12.5 (9/72)	1.6 (1/64)	4.0 (2/50)	
PNOS		18.9 (7/37)	19.4 (6/31)	10.5 (2/19)	
Poor (≤49)					
Total^a^		4.7 (17/362)	6.5 (20/308)	5.1 (12/236)	3.4 (5/147)
Schizophrenia		4.0 (10/253)	5.2 (11/213)	4.2 (7/167)	1.8 (2/113)
Schizophreniform disorder		5.6 (4/72)	7.8 (5/64)	4.0 (2/50)	7.7 (2/26)
PNOS		8.1 (3/37)	12.9 (4/31)	15.8 (3/19)	12.5 (1/8)
LAIA	9.7 (52)	18.3 (70)	21.7 (72)	22.0 (56)	25.5 (40)
Aripiprazole monohydrate once-monthly^b^	11.3 (6)/818.3 ± 246.7	27.1 (19)/543.2 ± 227.5	32.0 (23)/518.3 ± 213.1	51.8 (29)/499.1 ± 226.7	57.5 (23)/558.7 ± 254.6
Paliperidone palmitate once-monthly^b^	86.8 (46)/597.8 ± 242.1	72.9 (51)/550.1 ± 231.6	68.1 (49)/522.3 ± 217.6	48.2 (27)/502.2 ± 236.3	42.5 (17)/546.6 ± 247.5
Metabolic syndrome					
Total^a^		12.8 (36/281)	17.4 (44/253)	22.0 (40/182)	11.5 (3/26)
Schizophrenia		13.6 (26/191)	17.5 (29/166)	23.6 (29/123)	5.9 (1/17)
Schizophreniform disorder		12.7 (8/63)	20.0 (12/60)	19.1 (9/47)	28.6 (2/7)
PNOS		7.4 (2/27)	11.1 (3/27)	16.7 (2/12)	- (0/2)

*LAIA* Long-Acting Injectable Antipsychotics, *m* months, *PNOS* Psychotic disorder not otherwise specified.

^a^Total participants with schizophrenia, schizophreniform disorder or PNOS.

^b^% (*n*)/Chlorpromazine equivalent dose of LAIA.

**Table 4:** User rates (%) for mental health welfare center.

	Baseline	6 m	12 m	24 m	36 m
Candidate^a^	85.0 (464/546)	60.0 (235/392)	50.0 (171/343)	43.6 (114/261)	54.6 (88/161)
User	7.4 (40/546)	12.4 (49/392)	11.0 (38/343)	10.4 (26/261)	12.1 (19/161)

*m* months.

^a^Those who were rated as having SOFAS score ≤ 70.

A comparison of the demographic and clinical characteristics of subjects being followed up versus those that had dropped out revealed no significant differences, with the exception of education and intensity of suicidal ideation (Table S2). Stepwise logistic regression revealed that significant predictors for full recovery were log(DUP + 1) (odds ratio [OR] 0.534, 95% confidence intervals [CI] = 0.364–0.758; *p* = 0.001), FI (OR 2.262, 95% CI = 1.361–3.967; *p* = 0.003) and PAR intensity (OR, 1.231, 95% CI = 1.013–1.512; *p* = 0.040) (Table 5). Table S3 presents the results of predictors by univariate logistic regression. In subgroup analysis, significant predictors for full recovery in SZ were log(DUP + 1) (OR 0.455, 95% CI = 0.276–0.709; *p* = 0.001), PANSS-positive (OR 0.929, 95% CI = 0.865–0.991; *p* = 0.033) and FI (OR, 2.405, 95% CI = 1.346–4.643; *p* = 0.005) (Table S4). For SZFD and PNOS, there were no significant predictors (Tables S3–2 and S3–3).

**Table 5:** Predictors of full recovery at 3-year follow-up in patients with early stage psychosis^a^ (*n* = 157).

	OR	95% CI	*p*-value
Log(DUP + 1)	0.534	0.364–0.758	**0.001**
PANSS, negative	0.948	0.892–1.004	0.078
FI, mean	2.262	1.361–3.967	**0.003**
PAR, intensity	1.231	1.013–1.512	**0.040**

*CI* confidence interval, *DUP* duration of untreated psychosis, *FI* Family Intimacy, *OR* odds ratio, *PAR* Physical Activity Rating, *PNOS* Psychotic disorder not otherwise specified.

^a^Total participants with schizophrenia, schizophreniform disorder or PNOS. The bolded values are significantly different.

## Discussion

Psychotic disorders have huge effects in terms of disruption to personal psychosocial development, caregiver burden, and medical costs. To help patients with early-stage psychosis recover, it is critical to understand the disorder’s trajectory including remission, recovery, and relapse. The KEPS enabled us to investigate outcomes over a 3-year period and identify the predictors for a full recovery at year 3 in patients with early-stage psychosis.

We observed remission and full recovery rates of 69–80% and 23–40% in total subjects, respectively, over the 3-year follow-up period. One previous systematic review reported that the mean remission rate in FEP was 36%^9^, and another reported a rate of 58%^10^. In Asian populations, symptomatic remission rates at the 1- and 3-year follow-ups have been reported at 60%^13^ and 59%^14^, respectively. The most recent study reported that 70% of FEP patients achieved symptomatic remission within the first 24 months of treatment^19^. With regard to recovery rate, studies have reported 17% (9–20%) over 10 years in SZ^11^ and 38% (30–46%) with a mean follow-up of 7.2 years in FEP^10^. Overall, our findings on remission and full recovery are similar to or better than those of previous studies. We applied more strict criteria of symptom improvement for full recovery (≤2 on the eight items of the PANSS) compared to most earlier studies^10,19,23^, and used Andreasen et al.’s (2005)^62^ remission criteria (≤3 on the eight items of the PANSS). We were eager to compare our results with other studies using the same criteria as ours, but we could only find one study that reported a 9.9% recovery rate over a mean period of 10.2 years of follow-up^65^; notably, those authors applied 2 years of sustained improvement, and participants were at various stages of illness. Especially, it was of interest to observe higher remission and recovery rates in SZFD and PNOS compared to SZ at 6- and 12-month follow-ups. This finding is in line with our previous report^67^ and other studies^68^. However, remission and recovery rates in PNOS were lower compared to SZ at 24- and/or 36-month follow-ups. This may be due to higher drop-out rate in PNOS^67^ leaving more severe subjects to be followed up. Regardless of the various results among the studies, the key point of emphasis is the large gap between remission and recovery rates over a 3-year continuous period. Hence, it seems imperative to provide intensive psychosocial interventions to those who have achieved symptomatic remission but are still suffering from functional impairment.

With regard to drop-out rates, our results are in line with one systematic review^69^ that reported disengagement rates for FEP ranging from 20.5–40% across studies and concluded that about 30% of people disengage from treatment, despite ongoing therapeutic need. Given the importance of the continuity of care for FEP, our findings strongly suggest that hospital-based case management be provided to ensure successful engagement in treatment. The Korea Ministry of Health and Welfare has recently incorporated this into their 2020 comprehensive plan for mental health promotion. With regard to relapse rates, our rates are relatively low compared to those of other studies^30,70^. This may be due to the fact that we did not include those who had dropped out without relapse, but other studies have also calculated the relapse rate using the same method we used^71,72^. Alternatively, it may be related to the high percentage of good adherence in the present study. It should be noted, however, that adherence was calculated only for participants with an adherence rating. In other words, if we included ‘drop-out’ as poor adherence, the rates of good and poor adherence would be lower and higher than the current ones, respectively. Another possibility may be associated with the modest prescription rate of LAIA. The prescription rate of LAIA was roughly 18–26% in the present study, which is considerably higher than the 15.3% reported for East Asian countries^73^ and even the 3.3% rate reported for Korea^74^. Considering that LAIA acceptance rates have been reported at 32.7% for patients with FEP^75^ and that psychiatrists offered antipsychotic depot treatment to only 35% of their patients suffering from SZ or schizoaffective disorder^76^, there seems to be more room for LAIA. It is of interest that relapse rates in PNOS were relatively higher compared to SZ. Considering the high drop-out rate in PNOS, this highlights the special need to provide psychoeducation or compliance therapy to patients with early PNOS. Additionally, rates of users accessing community mental health welfare centers were low, even though the rates of eligible patients were relatively high (44–60%). This signifies that referral is not effective, possibly due to resistance among psychiatrists (low referral rates) or patients and their caregivers (fear of being exposed to new persons), or the facilities of the mental health welfare centers may be inadequate.

With regard to predictors of full recovery, significant factors were shorter DUP, FI and intensity of the PAR. Accumulating evidence indicates an association between a shorter DUP and FEP recovery^12,14,15,17,18^. The DUP can be reduced by early intervention^77^ and by a 2-week referral-to-treatment target for psychosis^78^. Considering that the DUP in the present study was 14.1 ± 26.3 months, more active awareness campaigns and the establishment of effective referral systems are required. Interestingly, to our knowledge, this is the first study to report the significant associations of FI and intensity of the PAR with recovery in early-stage psychosis. Two previous studies using qualitative interviews revealed that family plays a critical role in the recovery process^56,79^. Evidence also suggests that exercise positively affects brain abnormalities, cognitive function, social-role functioning, quality of life, symptomatology, and obesity in psychosis^80^. Therefore, it is not surprising that FI and physical activity have positive effects on recovery. Interestingly, frequency of PAR was not linked to recovery. In SZ, predictors were not different except the PANSS-positive which showed a negative association with recovery.

The present study had several limitations that should be considered. First, we did not include the drop-out in calculating remission, recovery, relapse (only drop-out without relapse not counted), and adherence. It is possible that we underestimated those rates, by assuming that the drop-out could be worst cases. We are planning to review the medical records of these patients through cooperation from the Health Insurance Review and Assessment Service. Second, we did not include variables affecting strongly outcomes such as premorbid functioning and cognition which may have affected the results. It is of note that our focus was on childhood trauma, rumination and lifestyle factors (diet and exercise), not already well-known factors. Third, as the definition of the early stage seems loose and may recruit heterogeneous subjects, findings should be interpreted cautiously. Fourth, we did not collect information on medication adherence from family members, which may have affected the validity of the adherence rating. Fifth, childhood trauma was evaluated on the self-rating scale, ETISR-SF. To enhance reliability, interview-based assessment is the preferred option. Despite these caveats, this study provides comprehensive results on clinical outcomes over a 3-year period in patients with early-stage psychosis. Especially, our findings highlight the importance of FI and physical exercise in improving clinical outcomes in patients with early-stage psychosis. In conclusion, we observed similar or better results in terms of remission, recovery, and relapse rates compared to other previous studies. Effective psychosocial intervention should be provided to shorten the gap between remission and recovery rates. More specifically, psychosocial intervention targeting DUP, family issues, and exercise would be helpful to enhance recovery.

## Supplementary information

2022-3 year outcome in early stage psychosis-Suppl-6

## Data Availability

The data used in this study can be acquired upon request. Because of restrictions based on privacy regulations and informed consent of participants, data cannot be made freely available in a public repository.

## Code availability

All relevant analysis scripts are available upon request.
