# Supplementary material for: Three-year outcomes and predictors for full recovery in patients with early-stage psychosis
Source: Schizophrenia (Heidelb). 2022 Oct 27;8(1):87. doi: 10.1038/s41537-022-00301-4 (PMC9613771; doi:10.1038/s41537-022-00301-4)
Supplement: Supplementary file 1 — 2022-3 year outcome in early stage psychosis-Suppl-6 [file 41537_2022_301_MOESM1_ESM.docx]

**Table S1.** Rates (%) of symptomatic remission and full recovery over 3-year follow-up

|  | **6 m (n=382)** | **12 m (n=332)** | **24 m (n=255)** | **36 m (n=157)** |
| --- | --- | --- | --- | --- |
| Symptomatic remission |  |  |  |  |
| Total^a^ | 73.6 (262/356) | 67.1 (206/307) | 78.5 (183/233) | 78.6 (121/154) |
| Schizophrenia | 70.2 (172/245) | 62.5 (130/208) | 77.4 (127/164) | 77.4 (89/115) |
| Schizophreniform disorder | 79.5 (58/73) | 77.6 (52/67) | 84.3 (43/51) | 80.0 (24/30) |
| PNOS | 84.2 (32/38) | 75.0 (24/32) | 72.2 (13/18) | 88.9 (8/9) |
| Full recovery |  |  |  |  |
| Total^a^ | 23.9 (85/355) | 25.3 (78/308) | 27.8 (65/234) | 39.0 (60/154) |
| Schizophrenia | 20.5 (50/244) | 18.8 (39/208) | 28.7 (47/164) | 38.3 (44/115) |
| Schizophreniform disorder | 32.9 (24/73) | 39.7 (27/68) | 27.5 (14/51) | 43.3 (13/30) |
| PNOS | 28.9 (11/38) | 37.5 (12/32) | 21.1 (4/19) | 33.3 (3/9) |

*PNOS* Psychotic disorder not otherwise specified, *m* months.

^a^Total participants with schizophrenia, schizophreniform disorder or PNOS.

Table S2. Demographic and baseline clinical characteristics of total participants followed up or dropped out over 3-year follow-up

|  | n | Overall (n=321) | Completers  (n=157) | Drop-out  (n=164) | *p*-value |
| --- | --- | --- | --- | --- | --- |
| Gender, male | 321 | 133 (41.4) | 64 (40.8) | 69 (42.1) | 0.901 |
| Age, years | 321 | 28.1±8.2 | 28.6±8.3 | 27.7±8.0 | 0.349 |
| Education | 321 |  |  |  | **0.009** |
| Elementary school |  | 2 (0.6) | 2 (1.3) | 0 (0) |  |
| Middle school |  | 134 (41.7) | 54 (34.4) | 80 (48.8) |  |
| University |  | 185 (57.6) | 101 (64.3) | 84 (51.2) |  |
| Type of medical insurance | 310 |  |  |  | 0.607 |
| Health insurance |  | 288 (92.9) | 139 (92.7) | 149 (93.1) |  |
| Near poor |  | 4 (1.3) | 1 (0.7) | 3 (1.9) |  |
| Medicaid |  | 18 (5.8) | 10 (6.7) | 8 (5) |  |
| Job type | 321 |  |  |  | 0.951 |
| Unemployment |  | 108 (33.6) | 54 (34.4) | 54 (32.9) |  |
| Non-professional |  | 193 (60.1) | 93 (59.2) | 100 (61) |  |
| Professional |  | 20 (6.2) | 10 (6.4) | 10 (6.1) |  |
| Family history of psychosis, yes | 320 | 35 (10.9) | 17 (10.8) | 18 (11) | 1.000 |
| Log(DUP+1) | 320 | 1.9±1.2 | 1.8±1.2 | 1.9±1.2 | 0.443 |
| DI, months | 321 | 20.9±25.7 | 18.8±21.4 | 22.9±29.1 | 0.152 |
| Diagnosis | 321 |  |  |  | 0.209 |
| Schizophrenia |  | 237 (73.8) | 117 (74.5) | 120 (73.2) |  |
| Schizophreniform disorder |  | 55 (17.1) | 30 (19.1) | 25 (15.2) |  |
| Psychotic disorder NOS |  | 29 (9) | 10 (6.4) | 19 (11.6) |  |
| Comorbid mental disorder, yes | 321 | 38 (11.8) | 16 (10.2) | 22 (13.4) | 0.471 |
| PNASS |  |  |  |  |  |
| Positive | 321 | 18.3±7.8 | 18.0±7.5 | 18.6±8.0 | 0.506 |
| Negative | 321 | 18.3±7.0 | 17.5±6.9 | 19.0±7.0 | 0.064 |
| General | 321 | 38.5±13.2 | 37.3±11.8 | 39.7±14.4 | 0.099 |
| Total | 321 | 75.1±25.5 | 72.8±23.5 | 77.3±27.1 | 0.117 |
| CDSS, total score | 319 | 5.2±4.8 | 5.3±4.9 | 5.1±4.8 | 0.652 |
| C-SSRS |  |  |  |  |  |
| Concreteness of suicidal ideation^a^ | 105 | 2.6±1.4 | 2.4±1.4 | 2.8±1.3 | 0.125 |
| Intensity of suicidal ideation^b^ | 104 | 2.5±1.2 | 2.2±1.0 | 2.7±1.2 | **0.016** |
| SOFAS | 321 | 55.0±13.6 | 55.4±14.3 | 54.7±13 | 0.653 |
| BCSS |  |  |  |  |  |
| Negative-self ^c^ | 309 | 1.7±0.8 | 1.7±0.8 | 1.7±0.8 | 0.903 |
| Negative-others^d^ | 309 | 1.8±0.7 | 1.8±0.7 | 1.8±0.7 | 0.414 |
| BFI-10, mean |  |  |  |  |  |
| Extraversion | 306 | 2.9±0.8 | 2.9±0.7 | 2.9±0.8 | 0.820 |
| Agreeableness | 306 | 3.4±0.7 | 3.4±0.7 | 3.3±0.8 | 0.271 |
| Conscientiousness | 306 | 3.1±0.7 | 3.1±0.8 | 3.0±0.7 | 0.136 |
| Neuroticism | 306 | 3.0±0.8 | 3.0±0.8 | 2.9±0.8 | 0.752 |
| Openness | 306 | 3.5±0.9 | 3.5±0.9 | 3.5±0.9 | 0.546 |
| BRS, mean | 307 | 2.8±0.8 | 2.8±0.7 | 2.8±0.8 | 0.919 |
| BS, total | 309 | 15.1±8.7 | 15.2±8.3 | 15.0±9.2 | 0.863 |
| DHQ | 303 |  |  |  | 0.650 |
| 20-49 |  | 47 (15.5) | 23 (14.8) | 24 (16.2) |  |
| 50-79 |  | 190 (62.7) | 101 (65.2) | 89 (60.1) |  |
| 80-100 |  | 66 (21.8) | 31 (20) | 35 (23.6) |  |
| ETISR-SF |  |  |  |  |  |
| General trauma | 308 | 1.8±2.0 | 1.7±1.9 | 1.8±2.2 | 0.421 |
| Physical abuse | 308 | 1.9±1.7 | 1.9±1.6 | 1.9±1.7 | 0.793 |
| Emotional abuse | 308 | 1.8±1.8 | 1.8±1.7 | 1.9±1.8 | 0.581 |
| Sexual abuse | 308 | 0.6±1.2 | 0.6±1.2 | 0.6±1.2 | 0.968 |
| Total | 308 | 6.1±5.1 | 6.0±4.9 | 6.3±5.4 | 0.668 |
| FACES-III, total | 272 | 61.3±13.1 | 61.4±12.3 | 61.1±14.1 | 0.846 |
| FI, mean | 271 | 3.7±0.9 | 3.8±0.9 | 3.6±0.9 | 0.070 |
| KmSWN, total | 305 | 56.0±16.7 | 56.1±17.0 | 56.0±16.4 | 0.979 |
| PAR |  |  |  |  |  |
| Frequency | 307 | 2.6±2.3 | 2.5±2.3 | 2.6±2.4 | 0.846 |
| Intensity | 307 | 1.8±1.9 | 1.9±1.9 | 1.7±1.8 | 0.405 |

*BCSS* Brief Core Schema Scales, *BFI-10* Big Five Inventory, *BRS* Brief Resilience Scale, *BS* Brooding scale, *CDSS* Calgary Depression Scale for Schizophrenia, *C-SSRS* Columbia-Suicide Severity Rating Scale, *DI* Duration of illness, *DHQ* Diet History Questionnaire, *DUP* duration of untreated psychosis, *ETISR-SF* Early Trauma Inventory Self Report-Short Form, *FACES-III* Family Adaptability and Cohesion Evaluation Scales III, *FI* Family Intimacy, *KmSWN* Korean modification of Subjective Well-being Under Neuroleptics-Short Form, *NOS* not otherwise specified, *PANSS* Positive and Negative Syndrome Scale, *PAR* Physical Activity Rating, *SOFAS* Social and Occupational Functioning Assessment Scale.

Data are presented as mean and standard deviation or number (%).

^a^Highest score on the suicidal ideation of the Columbia-Suicide Severity Rating Scale (C-SSRS).

^b^Average of the scores on frequency and duration for intensity of ideation of the C-SSRS.

^c^Average of the score of negative-self schema and reversed score of positive-self schema of the Brief Core Schema Scales (BCSS).

^d^Average of the score of negative-others schema and reversed score of positive-others schema of the BCSS.

Table S3. Predictors of full recovery at 3-year follow-up in patients with early stage psychosis^a^ (n=157)

|  | OR | 95% CI | *p*-value |
| --- | --- | --- | --- |
| Gender, male | 0.664 | 0.341-1.277 | 0.224 |
| Age, years | 0.980 | 0.941-1.019 | 0.324 |
| Education |  |  |  |
| Elementary school | 1.000 (ref) | | |
| Middle school | 0.421 | 0.016-11.086 | 0.550 |
| University | 0.836 | 0.032-21.542 | 0.900 |
| Type of medical insurance |  |  |  |
| Health insurance | 1.000 (ref) | | |
| Near poor | - | - | - |
| Medicaid | 0.360 | 0.053-1.500 | 0.206 |
| Job type |  |  |  |
| Unemployment | 1.000 (ref) | | |
| Non-professional | 1.038 | 0.524-2.079 | 0.915 |
| Professional | 1.571 | 0.393-6.297 | 0.513 |
| Family history of psychosis, yes | 0.589 | 0.179-1.682 | 0.344 |
| Log(DUP+1) | 0.539 | 0.386-0.733 | **0.000** |
| Duration of illness, months | 0.969 | 0.946-0.988 | **0.004** |
| Diagnosis |  |  |  |
| Schizophrenia |  | 1.000 (ref) |  |
| Schizophreniform disorder | 1.400 | 0.618-3.148 | 0.415 |
| Psychotic disorder NOS | 1.067 | 0.261-3.940 | 0.924 |
| Comorbid mental disorder, yes | 0.463 | 0.125-1.404 | 0.201 |
| PNASS |  |  |  |
| Positive | 0.964 | 0.921-1.007 | 0.102 |
| Negative | 0.941 | 0.892-0.988 | **0.020** |
| General | 0.981 | 0.952-1.008 | 0.175 |
| Total | 0.986 | 0.971-1.000 | 0.058 |
| CDSS, total score | 0.940 | 0.874-1.006 | 0.086 |
| C-SSRS |  |  |  |
| Concreteness of suicidal ideation | 0.759 | 0.453-1.197 | 0.257 |
| Intensity of suicidal ideation | 1.166 | 0.646-2.107 | 0.604 |
| SOFAS | 1.020 | 0.997-1.044 | 0.098 |
| BCSS, mean |  |  |  |
| Self | 0.686 | 0.444-1.039 | 0.080 |
| Others | 0.639 | 0.393-1.016 | 0.063 |
| BFI-10, mean |  |  |  |
| Extraversion | 1.266 | 0.821-1.974 | 0.290 |
| Agreeableness | 1.247 | 0.805-1.959 | 0.328 |
| Conscientiousness | 1.186 | 0.773-1.839 | 0.438 |
| Neuroticism | 1.150 | 0.753-1.770 | 0.518 |
| Openness | 1.237 | 0.858-1.797 | 0.257 |
| BRS, mean | 1.481 | 0.945-2.373 | 0.092 |
| BS, total | 0.983 | 0.945-1.021 | 0.373 |
| DHQ |  |  |  |
| 20-49 | 1.000 (ref) | | |
| 50-79 | 1.335 | 0.529-3.580 | 0.549 |
| 80-100 | 1.184 | 0.387-3.725 | 0.768 |
| ETISR-SF |  |  |  |
| General trauma | 0.835 | 0.686-1.001 | 0.060 |
| Physical abuse | 0.800 | 0.645-0.982 | **0.036** |
| Emotional abuse | 0.904 | 0.747-1.087 | 0.288 |
| Sexual abuse | 0.998 | 0.751-1.305 | 0.986 |
| Total | 0.938 | 0.873-1.004 | 0.073 |
| FACES-III, total | 1.044 | 1.014-1.079 | **0.006** |
| FI, mean | 2.161 | 1.401-3.486 | **0.001** |
| KmSWN, total | 1.027 | 1.007-1.050 | **0.010** |
| PAR |  |  |  |
| Frequency | 1.056 | 0.920-1.213 | 0.439 |
| Intensity | 1.250 | 1.057-1.491 | **0.010** |
| Metabolic syndrome | 1.451 | 0.474-4.358 | 0.504 |

*BCSS* Brief Core Schema Scales, *BFI-10* Big Five Inventory, *BRS* Brief Resilience Scale, *BS* Brooding scale, *CDSS* Calgary Depression Scale for Schizophrenia, *CI* confidence interval, *C-SSRS* Columbia-Suicide Severity Rating Scale, *DI* Duration of illness, *DHQ* Diet History Questionnaire, *DUP* duration of untreated psychosis, *ETISR-SF* Early Trauma Inventory Self Report-Short Form, *FACES-III* Family Adaptability and Cohesion Evaluation Scales III, *FI* Family Intimacy, *KmSWN* Korean modification of Subjective Well-being Under Neuroleptics-Short Form, *NOS* not otherwise specified, *OR* odds ratio, *PANSS* Positive and Negative Syndrome Scale, *PAR* Physical Activity Rating, *SOFAS* Social and Occupational Functioning Assessment Scale.

Values in bold: p<0.05; values underlined: 0.05≤p≤0.1.

^a^Include schizophrenia, schizophreniform disorder and PNOS.

Table S3-1. Predictors of full recovery at 3-year follow-up in patients with early stage schizophrenia (n=117)

|  | OR | 95% CI | *p*-value |
| --- | --- | --- | --- |
| Gender, male | 0.718 | 0.332-1.527 | 0.392 |
| Age, years | 0.978 | 0.931-1.025 | 0.365 |
| Education |  |  |  |
| Elementary school | 1.000 (ref) | | |
| Middle school | 0.379 | 0.014-10.15 | 0.506 |
| University | 0.786 | 0.03-20.369 | 0.866 |
| Type of medical insurance |  |  |  |
| Health insurance | 1.000 (ref) | | |
| Near poor | - | - | - |
| Medicaid | - | - | - |
| Job type |  |  |  |
| Unemployment | 1.000 (ref) | | |
| Non-professional | 1.040 | 0.472-2.330 | 0.923 |
| Professional | 5.200 | 0.605-110.452 | 0.169 |
| Family history of psychosis, yes | 0.443 | 0.095-1.548 | 0.236 |
| Log(DUP+1) | 0.464 | 0.305-0.676 | **0.000** |
| Duration of illness, months | 0.965 | 0.938-0.987 | **0.005** |
| Comorbid mental disorder, yes | 0.372 | 0.054-1.572 | 0.225 |
| PNASS |  |  |  |
| Positive | 0.928 | 0.875-0.979 | **0.009** |
| Negative | 0.944 | 0.888-0.998 | 0.051 |
| General | 0.962 | 0.927-0.994 | **0.029** |
| Total | 0.978 | 0.960-0.995 | **0.014** |
| CDSS, total score | 0.927 | 0.846-1.004 | 0.077 |
| C-SSRS |  |  |  |
| Concreteness of suicidal ideation | 0.630 | 0.339-1.064 | 0.106 |
| Intensity of suicidal ideation | 0.894 | 0.416-1.810 | 0.759 |
| SOFAS | 1.023 | 0.997-1.052 | 0.091 |
| BCSS, mean |  |  |  |
| Self | 0.737 | 0.453-1.171 | 0.204 |
| Others | 0.620 | 0.353-1.055 | 0.085 |
| BFI-10, mean |  |  |  |
| Extraversion | 1.135 | 0.705-1.843 | 0.602 |
| Agreeableness | 1.005 | 0.611-1.662 | 0.984 |
| Conscientiousness | 1.106 | 0.689-1.791 | 0.678 |
| Neuroticism | 1.257 | 0.787-2.040 | 0.342 |
| Openness | 1.182 | 0.779-1.806 | 0.434 |
| BRS, mean | 1.571 | 0.932-2.735 | 0.097 |
| BS, total | 0.980 | 0.938-1.024 | 0.373 |
| DHQ |  |  |  |
| 20-49 | 1.000 (ref) | | |
| 50-79 | 1.745 | 0.584-5.936 | 0.338 |
| 80-100 | 1.200 | 0.316-4.846 | 0.790 |
| ETISR-SF |  |  |  |
| General trauma | 0.711 | 0.537-0.907 | **0.010** |
| Physical abuse | 0.778 | 0.608-0.982 | **0.039** |
| Emotional abuse | 0.870 | 0.699-1.072 | 0.199 |
| Sexual abuse | 0.983 | 0.674-1.392 | 0.923 |
| Total | 0.909 | 0.830-0.988 | **0.031** |
| FACES-III, total | 1.042 | 1.008-1.081 | **0.018** |
| FI, mean | 2.365 | 1.416-4.217 | **0.002** |
| KmSWN, total | 1.020 | 0.998-1.044 | 0.081 |
| PAR |  |  |  |
| Frequency | 1.058 | 0.899-1.245 | 0.498 |
| Intensity | 1.225 | 1.016-1.491 | **0.036** |
| Metabolic syndrome | 1.190 | 0.324-4.104 | 0.783 |

*BCSS* Brief Core Schema Scales, *BFI-10* Big Five Inventory, *BRS* Brief Resilience Scale, *BS* Brooding scale, *CDSS* Calgary Depression Scale for Schizophrenia, *CI* confidence interval, *C-SSRS* Columbia-Suicide Severity Rating Scale, *DI* Duration of illness, *DHQ* Diet History Questionnaire, *DUP* duration of untreated psychosis, *ETISR-SF*, Early Trauma Inventory Self Report-Short Form, *FACES-III* Family Adaptability and Cohesion Evaluation Scales III, *FI* Family Intimacy, *KmSWN* Korean modification of Subjective Well-being Under Neuroleptics-Short Form, *NOS* not otherwise specified, *OR* odds ratio, *PANSS* Positive and Negative Syndrome Scale, *PAR* Physical Activity Rating, *SOFAS* Social and Occupational Functioning Assessment Scale.

Table S3-2. Predictors of full recovery at 3-year follow-up in patients with early stage schizophreniform disorder (n=30)

|  | OR | 95% CI | *p*-value |
| --- | --- | --- | --- |
| Gender, male | 0.400 | 0.081-1.764 | 0.237 |
| Age, years | 0.990 | 0.911-1.071 | 0.799 |
| Education |  |  |  |
| Elementary school | 1.000 (ref) | | |
| Middle school | - | - | - |
| University | 1.400 | 0.322-6.378 | 0.654 |
| Type of medical insurance |  |  |  |
| Health insurance | 1.000 (ref) | | |
| Near poor | - | - | - |
| Medicaid | - | - | - |
| Job type |  |  |  |
| Unemployment | 1.000 (ref) | | |
| Non-professional | 1.687 | 0.351-8.783 | 0.517 |
| Professional | 0.750 | 0.029-10.779 | 0.835 |
| Family history of psychosis, yes | - | - | - |
| Log(DUP+1) | 1.475 | 0.348-6.774 | 0.597 |
| Duration of illness, months | 1.027 | 0.885-1.211 | 0.712 |
| Comorbid mental disorder, yes | 1.167 | 0.124-10.971 | 0.886 |
| PNASS |  |  |  |
| Positive | 1.086 | 0.980-1.224 | 0.135 |
| Negative | 0.913 | 0.787-1.040 | 0.191 |
| General | 1.054 | 0.984-1.145 | 0.158 |
| Total | 1.020 | 0.982-1.064 | 0.318 |
| CDSS, total score | 0.984 | 0.841-1.147 | 0.837 |
| C-SSRS |  |  |  |
| Concreteness of suicidal ideation | 2.498 | 0.721-14.423 | 0.186 |
| Intensity of suicidal ideation | - | - | - |
| SOFAS | 0.981 | 0.928-1.035 | 0.496 |
| BCSS, mean |  |  |  |
| Self | 0.678 | 0.227-1.876 | 0.461 |
| Others | 0.688 | 0.222-1.952 | 0.488 |
| BFI-10, mean |  |  |  |
| Extraversion | 2.981 | 0.816-13.936 | 0.121 |
| Agreeableness | 2.413 | 0.826-8.310 | 0.126 |
| Conscientiousness | 1.629 | 0.474-6.303 | 0.447 |
| Neuroticism | 0.683 | 0.192-2.111 | 0.514 |
| Openness | 1.315 | 0.520-3.511 | 0.565 |
| BRS, mean | 0.889 | 0.284-2.648 | 0.830 |
| BS, total | 0.994 | 0.905-1.091 | 0.896 |
| DHQ |  |  |  |
| 20-49 | 1.000 (ref) | | |
| 50-79 | 0.818 | 0.084-7.958 | 0.855 |
| 80-100 | 1.000 | 0.056-17.887 | 1.000 |
| ETISR-SF |  |  |  |
| General trauma | 1.080 | 0.785-1.514 | 0.634 |
| Physical abuse | 0.876 | 0.506-1.453 | 0.611 |
| Emotional abuse | 1.050 | 0.654-1.692 | 0.837 |
| Sexual abuse | 1.018 | 0.625-1.650 | 0.938 |
| Total | 1.010 | 0.880-1.160 | 0.881 |
| FACES-III, total | 1.039 | 0.967-1.128 | 0.312 |
| FI, mean | 2.031 | 0.586-8.326 | 0.281 |
| KmSWN, total | 1.049 | 0.999-1.114 | 0.079 |
| PAR |  |  |  |
| Frequency | 1.013 | 0.742-1.382 | 0.934 |
| Intensity | 1.295 | 0.853-2.086 | 0.242 |
| Metabolic syndrome | - | - | - |

*BCSS* Brief Core Schema Scales, *BFI-10* Big Five Inventory, *BRS* Brief Resilience Scale, *BS* Brooding scale, *CDSS* Calgary Depression Scale for Schizophrenia, *CI* confidence interval, *C-SSRS* Columbia-Suicide Severity Rating Scale, *DI* Duration of illness, *DHQ* Diet History Questionnaire, *DUP* duration of untreated psychosis, *ETISR-SF* Early Trauma Inventory Self Report-Short Form, *FACES-III* Family Adaptability and Cohesion Evaluation Scales III, *FI* Family Intimacy, *KmSWN* Korean modification of Subjective Well-being Under Neuroleptics-Short Form, *NOS* not otherwise specified, *OR* odds ratio, *PANSS* Positive and Negative Syndrome Scale, *PAR* Physical Activity Rating, *SOFAS* Social and Occupational Functioning Assessment Scale.

Table S3-3. Predictors of full recovery at 3-year follow-up in patients with early stage PNOS (n=10)

|  | OR | 95% CI | *p*-value |
| --- | --- | --- | --- |
| Gender, male | 1.667 | 0.052-54.457 | 0.748 |
| Age, years | 0.929 | 0.697-1.144 | 0.526 |
| Education |  |  |  |
| Elementary school | 1.000 (ref) | | |
| Middle school | - | - | - |
| University | - | - | - |
| Type of medical insurance |  |  |  |
| Health insurance | 1.000 (ref) | | |
| Near poor | - | - | - |
| Medicaid | - | - | - |
| Job type |  |  |  |
| Unemployment | 1.000 (ref) | | |
| Non-professional | 0.167 | 0.003-3.748 | 0.287 |
| Professional | 0.250 | 0.005-6.562 | 0.423 |
| Family history of psychosis, yes | 0.667 | 0.024-10.856 | 0.779 |
| Log(DUP+1) | 0.111 | 0.001-1.007 | 0.205 |
| Duration of illness, months | 0.947 | 0.786-1.095 | 0.485 |
| Comorbid mental disorder, yes | - | - | - |
| PNASS |  |  |  |
| Positive | 0.886 | 0.582-1.257 | 0.511 |
| Negative | 0.901 | 0.517-1.453 | 0.671 |
| General | 0.965 | 0.770-1.184 | 0.730 |
| Total | 0.962 | 0.825-1.095 | 0.563 |
| CDSS, total score | 0.912 | 0.619-1.273 | 0.597 |
| C-SSRS |  |  |  |
| Concreteness of suicidal ideation | - | - | - |
| Intensity of suicidal ideation | 0.203 | 0.001-2.481 | 0.339 |
| SOFAS | 1.207 | 1.026-1.717 | 0.121 |
| BCSS, mean |  |  |  |
| Self | 0.071 | 0-1.463 | 0.184 |
| Others | 1.147 | 0.082-16.88 | 0.915 |
| BFI-10, mean |  |  |  |
| Extraversion | 1.798 | 0.115-54.847 | 0.684 |
| Agreeableness | - | - | - |
| Conscientiousness | 1.415 | 0.155-14.911 | 0.748 |
| Neuroticism | 0.841 | 0.053-12.794 | 0.895 |
| Openness | 1.852 | 0.428-11.867 | 0.436 |
| BRS, mean | 3.181 | 0.466-72.889 | 0.328 |
| BS, total | 0.974 | 0.766-1.204 | 0.802 |
| DHQ |  |  |  |
| 20-49 | 1.000 (ref) | | |
| 50-79 | 0.250 | 0.005-10.212 | 0.442 |
| 80-100 | 2.000 | 0.042-120.28 | 0.711 |
| ETISR-SF |  |  |  |
| General trauma | 1.083 | 0.435-2.755 | 0.858 |
| Physical abuse | 1.119 | 0.381-3.584 | 0.833 |
| Emotional abuse | 1.299 | 0.455-4.560 | 0.616 |
| Sexual abuse | 0.847 | 0.164-3.031 | 0.801 |
| Total | 1.047 | 0.724-1.562 | 0.801 |
| FACES-III, total | 1.116 | 0.914-1.473 | 0.327 |
| FI, mean | 1.214 | 0.346-4.763 | 0.757 |
| KmSWN, total | 1.888 | 1.101-20.937 | 0.241 |
| PAR |  |  |  |
| Frequency | 1.217 | 0.682-2.425 | 0.503 |
| Intensity | 2.152 | 0.821-11.754 | 0.211 |
| Metabolic syndrome | - | - | - |

*BCSS* Brief Core Schema Scales, *BFI-10* Big Five Inventory, *BRS* Brief Resilience Scale, *BS* Brooding scale, *CDSS* Calgary Depression Scale for Schizophrenia, *CI* confidence interval, *C-SSRS* Columbia-Suicide Severity Rating Scale, *DI* Duration of illness, *DHQ* Diet History Questionnaire, *DUP* duration of untreated psychosis, *ETISR-SF* Early Trauma Inventory Self Report-Short Form, *FACES-III* Family Adaptability and Cohesion Evaluation Scales III, *FI* Family Intimacy, *KmSWN* Korean modification of Subjective Well-being Under Neuroleptics-Short Form, *NOS* not otherwise specified, *OR* odds ratio, *PANSS* Positive and Negative Syndrome Scale, *PAR* Physical Activity Rating, *SOFAS* Social and Occupational Functioning Assessment Scale.

Table S4. Predictors of full recovery at 3-year follow-up in patients with early stage schizophrenia (n= 117)

|  | **OR** | **95% CI** | ***p*-value** |
| --- | --- | --- | --- |
| Log(DUP+1) | 0.455 | 0.276-0.709 | **0.001** |
| PANSS, positive | 0.929 | 0.865-0.991 | **0.033** |
| FI, mean | 2.405 | 1.346-4.643 | **0.005** |

*CI* confidence interval, *DUP* duration of untreated psychosis, *FI* Family Intimacy, *OR* odds ratio.
